# Rapid intraoperative amplicon sequencing of CNS tumor markers

**DOI:** 10.1016/j.csbj.2024.11.007

**Published:** 2024-11-08

**Authors:** Maximilian Evers, Björn Brändl, Christian Rohrandt, Carolin Kubelt-Kwamin, Franz-Josef Müller, Dominik Danso, André Maicher, Gaojianyong Wang, Sönke Friedrichsen, Stephan Kolkenbrock

**Affiliations:** a altona Diagnostics GmbH, Hamburg, Germany; b Institute of Biology and Biotechnology of Plants, University of Münster, Münster, Germany; c Department of Psychiatry and Psychotherapy, Christian-Albrechts-Universität zu Kiel, Kiel, Germany; d Department of Neurosurgery, University Medical Center Schleswig-Holstein UKSH, Campus Kiel, Kiel, Germany; e Department of Genome Regulation, Max Planck Institute for Molecular Genetics, Berlin, Germany

**Keywords:** CNS tumors, Amplicon sequencing, Tumor markers, Nanopore, Intraoperative

## Abstract

Currently, central nervous system tumors are diagnosed with an integrated diagnostic approach that combines histopathological examination with molecular genetic profiling, which requires days to weeks to achieve a precise and informative classification of CNS tumors. This study demonstrates the feasibility of rapid multiplex amplicon nanopore sequencing for identifying critical mutations relevant to molecular stratification of brain tumors within the timeframe of standard resection surgery. Utilizing live analysis of nanopore sequencing data, we evaluated the brain tumor-associated molecular markers IDH1 R132, IDH2 R172, *pTERT* C228 and C250, H3F3A K27 and G34, Hist1H3B K27, and BRAF V600. Our method achieved a turnaround time of 105 min at the point-of-care from receipt of a tumor biopsy to result with the potential to impact surgical strategy. Our approach can be integrated with recently developed DNA methylation-based diagnostic classification systems, corroborating diagnoses and even further specifying tumor grades, thus enabling a multimodal diagnostic intraoperative assessment of CNS malignancies.

## Graphical Abstract

**Figure:**
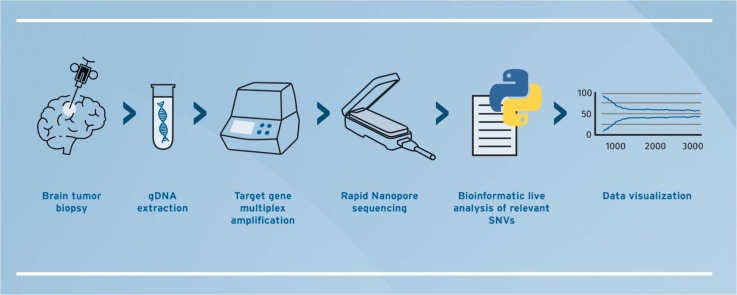

## 1 Main

### 1.1 Introduction

Tumors of the central nervous system (CNS) present significant diagnostic and treatment challenges. The World Health Organization (WHO) classifies CNS tumors using a multidimensional approach that includes histopathological examination, gene mutations, structural genomic variants, and epigenetic changes [1]. Conventional methods, such as real-time PCR [2], PCR from bisulfite-converted DNA [3], next-generation sequencing (NGS) [4], and microarray analysis of global DNA methylation patterns, have been instrumental in identifying tumor markers in preclinical research and have entered clinical practice; however, these methods often require days to weeks for results, potentially delaying treatment decisions.

Recent technological advances have aimed to reduce diagnosis times significantly. Intraoperative RT PCR-based identification of *IDH1* and *pTERT* mutations was demonstrated in 2015 [5]. Same-day amplicon sequencing of tumor markers was first shown in 2017 [4] and intraoperative global DNA methylation analysis and classification were first performed in 2021 [6].

Nanopore sequencing has been proposed due to its ability to natively call methylation during sequencing and the rapid acquisition and live processing of sequence data it provides [7]. The presented approach will focus on the increased speed of direct DNA sequencing to call diagnostically relevant single nucleotide variants (SNVs) during surgery. Knowledge of the underlying mutations gained through sequencing may obviate the need for further histologic analysis of these markers and potentially improve patient outcomes through rational adaptation of surgical strategies.

Increasingly, tumor marker analysis focuses on reducing the time-to-results to an intraoperative timeframe. Intraoperative diagnosis is considered advantageous for the potential adjustment of surgical strategies during brain tumor resections. This study leverages rapid multiplex amplicon nanopore sequencing to identify critical mutations directly during surgery. Nanopore sequencing has emerged as a prominent tool due to its ability to perform live processing and rapid data acquisition. This technology facilitates the identification of critical mutations in real time, offering a potential improvement in intraoperative diagnostic efficiency [7]. As the duration of a typical brain tumor surgery is approximately 3 h [8], we aimed for a time-to-results of the developed approach within this timeframe.

Mutations highlighted by the WHO2021 classification were examined for relevant tumor markers to aid surgeons with the assessment of the potential extent of resection (EOR). These tumor markers include IDH1 R132, IDH2 R172, *pTERT* C228 and C250, H3F3A K27/G34, Hist1H3B K27, and BRAF V600. [9].

Mutations in IDH1 and IDH2 are linked to DNA hypermethylation and impact overall survival (OS) across diverse tumor types [10]. A greater extent of resection generally leads to improved survival, with as little as 78 % EOR showing significant benefits [11]. Specific tumor types, including IDH-wildtype glioblastoma (GBM) and IDH-mutant astrocytoma, showed survival benefits after gross total resection [10], [12]. Conversely, diffuse midline gliomas harboring H3F3A K27M mutations are deemed incurable, emphasizing the importance of prioritizing quality-of-life preservation [13]. Other CNS-malignancies have been associated with K27M mutations, as well [14], [15]. The predominant BRAF mutation, V600E, is frequently found in pediatric low-grade astrocytomas, pleomorphic xanthoastrocytomas, epithelioid glioblastomas, and other tumor types. Research indicates that BRAF-targeted therapy using BRAF inhibitors can extend disease control in tumors with the V600E mutation [16]. Mutations in *pTERT* C228T and C250T are located in the noncoding promoter region of the telomerase reverse transcriptase (*TERT*) gene and form *de novo* transcription factor-binding sites involved in the transcriptional activation of *TERT* [17], leading to cell line immortality and proliferation due to the upregulation of *TERT* expression. Overall survival is impacted by *TERT* overexpression [18] and GBM patients with *pTERT* mutations have shorter OS than wild-type *pTERT* patients, with median OS times of 11 and 20 months, respectively [19].

Tumor purity is a prognostic factor in glioma, with lower purity associated with poorer prognosis [20]. Therefore, a low limit of detection for variant allele frequency (VAF) is advantageous for diagnostic tests aimed at assessing genetic CNS tumor markers.

In this study, we aimed to demonstrate the feasibility and potential clinical impact of rapid multiplex amplicon nanopore sequencing for intraoperative decision-making, focusing on mutations identified by the WHO 2021 classification as critical for CNS tumor diagnosis and treatment planning.

## 2 Results

### 2.1 Plasmid-derived amplicon sequencing

**Fig. 1:**
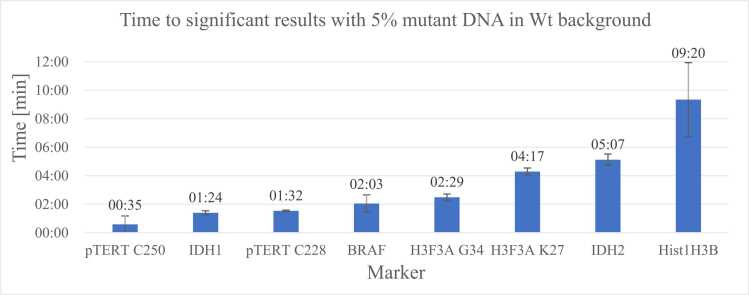
– Time necessary to identify significant (t-test; p ≤ 0.05) differences between 0 % VAF and 5 % VAF (Variants analyzed / time ± standard deviation: pTERT C250T / 00:35 ± 00:35 min, IDH1 R132H / 01:24 ± 0:09 min, pTERT C228T / 01:32 ± 0:03 min, BRAF V600E / 02:03 ± 00:36 min, H3F3A G34R / 02:29 ± 00:14 min, H3F3A K27M / 04:17 ± 00:15 min, IDH2 R172G / 05:07 ± 00:24 min, Hist1H3B K27M / 09:20 ± 02:36 min) depending on tumor markers on an ONT R9.4.1 Flongle flow cell using an SQK-RAD004 library. n = 3.

To establish amplification systems with highly controllable parameters, the amplification of target genes was performed on plasmids to generate the correct input sequences for library preparation. The expected amplicon size was confirmed via agarose gel electrophoresis before sequencing. A mixture of two plasmids was created for each marker. One plasmid carried a wild-type marker sequence, while the other carried a mutant variant. With this approach, we simulated heterozygosity and different variant allele frequencies (VAFs). These plasmid mixtures were used as the template for multiplex PCR, after which a VAF of 5 % was reliably detected for all markers during the first minutes of sequencing, revealing a surprisingly low limit of detection with this artificial system (Fig. 1).

### 2.2 gDNA-derived amplicon sequencing

The tested systems were then used to amplify tumor markers from patient samples. A total of 22 qualitative sequencing runs (with different amounts of analyzed markers) were performed on 17 different patient samples. All analyses were performed using a panel of IDH1, IDH2, pTERT C228 and C250. H3F3A K27 and G34 were additionally used in 16 of these 22 panels and 10 of those panels were also complemented with Hist1H3B K27 and BRAF V600. Where possible, previously collected tumor marker data was compared to the results obtained during amplicon sequencing. In total 30 marker instances were previously assessed and correctly identified via amplicon sequencing (Supplementary Figure 1).

**Fig. 2:**
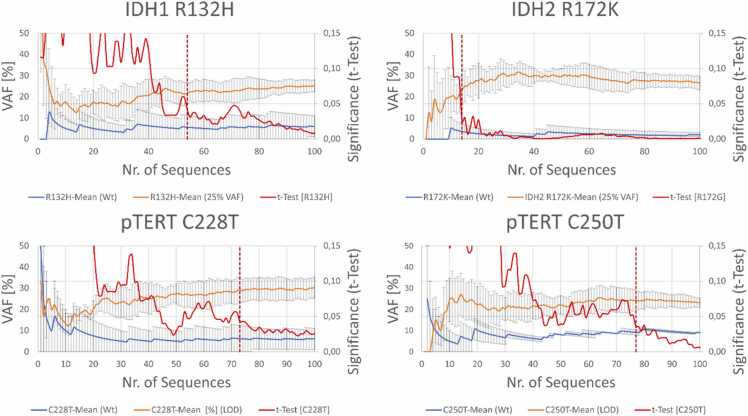
– Differences in the variant allele fraction (VAF) of four markers (IDH1, IDH2, pTERT C228, and pTERT C250) between sequences from wild-type genes (blue; n = 2) and 50 % diluted heterozygously mutated genes (orange; n = 3). The red solid line indicates the continuous significance of the difference between the two datasets. The dotted vertical line indicates the threshold number of sequences until a p-value of ≤ 0.05 was reached. Sequencing was performed on R9.4.1 Flongle flow cells.

The number of sequences necessary for statistically significant (p < 0.05, t-test) differentiation between wild-type and variant tumor markers was investigated for *IDH1, IDH2, pTERT* C228, and *pTERT* C250, as patient material with confirmed mutations was available. Genomic DNA isolated from tumor tissue was used as a template for multiplex PCR. The presence of tumor marker variations was confirmed through Sanger sequencing. Heterozygous IDH1 R132H and *pTERT* C228T mutations have been identified in sample IEG37, whereas heterozygous IDH2 R172K and *pTERT* C250T mutations have been identified in sample IEG42 (Supplementary Figure 2). Furthermore, these tumor samples were classified using recently published Sturgeon software [7] based on sparse whole genome methylation sequencing. Both samples were classified as oligodendrogliomas carrying an IDH mutation (Supplementary Figure 3, 4). The patient samples were mixed to dilute the heterozygous mutations of both samples. Using a dilution of 1:1 (gDNA concentration, determined via NanoDrop measurement), we could reliably identify known tumor marker variants with an appropriate VAF of ∼25 % (Fig. 2), demonstrating sensitivity even in cases of low tumor purity.

Considering the 25 % VAF for IDH1, IDH2, *pTERT* C228 and pTERT C250, significant (t-test; p ≤ 0.05) differences between Wt samples and tumor marker samples were identified with fewer than 80 sequences per marker (Sequencing performed on a Flongle R9.4.1 flow cell, IDH1 – 45 Seq, 00:45 ± 00:03 min; IDH2 – 14 Seq, 00:18 ± 00:12 min; pTERT C228 – 73 Seq, 38:30 ± 17:41 min; pTERT C250 – 77 Seq, 37:34 ± 18:58 min; Fig. 2).

### 2.3 Clinical Demonstrator

**Fig. 3:**
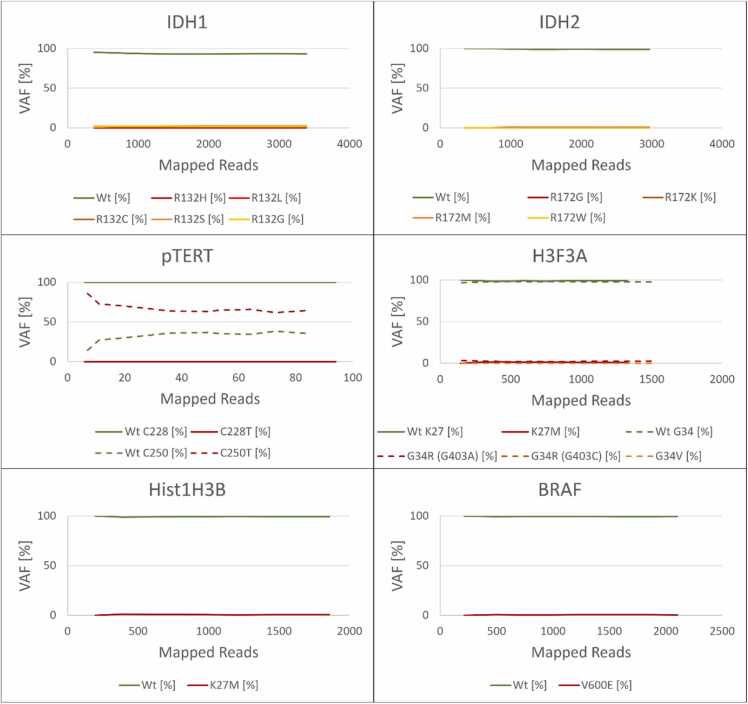
– Sequencing results of a clinical demonstrator after 20 min of sequencing. The variant allele fraction (VAF) of each analyzed marker variant is shown and plotted as a continuous graph. A pTERT C250T mutation was detected. Sequencing was performed on R9.4.1 MinION flow cells.

An early clinical demonstrator was performed using a marker panel without H3F3A, HistH3B and BRAF (Supplementary Figure 5, Supplementary Table 1). During workflow optimization these markers were included, and the corresponding clinical demonstrator is shown below. A tumor tissue sample was obtained at UKSH Kiel during tumor resection surgery. Isolation of gDNA from this biopsy was performed within 20 min and yielded a concentration of 50 ng/µL. For multiplex PCR, a 1:10 dilution (5 ng/µL) in nuclease-free H_2_O was used as the template, and all established amplification systems were used. After 20 min of sequencing using a MinION R10.4.1 flow cell, the preestablished read-amount thresholds for *IDH1*, *IDH2*, *pTERT* C228, and *pTERT* C250 were reached. A *pTERT* C250T mutation was identified (of 85 *pTERT* C250 reads, 55 reads or ∼65 % contained a C250T substitution; Fig. 3). Other marker variants were detected in negligible amounts (Supplementary Table 2). The sum of all steps yielded a time-to-results of 105 min beginning with the reception of the tumor tissue, including 20 min of gDNA extraction, 65 min of PCR plus library prep, and 20 min of sequencing (Supplementary Figure 6), generating results within 2 h after reception of the tumor tissue.

## 3 Discussion

Here, we demonstrate the feasibility of patient-agnostic, intraoperative amplicon-based nanopore sequencing of CNS tumor markers. As the treatment of CNS tumors is heavily dependent on the combination of its underlying mutations, a faster time-to-results might enable subsequently adjusted therapeutic strategies for a greater overall survival [21]. With the results of multiplex sequencing after 20 min and real-life time-to-results after delivery of the tumor sample of < 2 h, the presented method can identify a defined set of tumor markers well within the typical duration of a brain tumor resection surgery, potentially allowing the adaptation of the surgical strategy. Our amplicon sequencing clinical demonstrator was not performed on site and therefore required the storage of the extracted gDNA at −80 °C. This step was not considered when calculating the time-to-results, as it is unnecessary when performing the protocol at the point-of-care.

Prospectively, the marker panel chosen in this study can be modified to include a desired number of markers by adding or changing the amplification systems during multiplex PCR. However, the time-to-results should be critically reassessed in such cases. A potential limit of PCR efficacy may be approached with an increasing number of amplification systems, and thus, the cross-reactivity of systems is more challenging to avoid. These limitations can be addressed by running separate PCRs for each amplification system or multiplex subsets of amplification systems and pooling the purified amplicons prior to library preparation. While this approach requires more hands-on time, it ensures a more controlled library input.

Nanopore sequencing allows for adaptive sampling, a technique in which the generated sequence is mapped to a given target. If the generated sequence does not match the target sequence, the polarity of the flow cell is reversed and the DNA strand is ejected from the pore. This can reduce the need for pre-amplification of target genes. However, we found this approach counterproductive for our setup, as the high amount of background gDNA in a whole genome library prevents the acquisition of target sequences in a reasonable time frame.

Low tumor purity is correlated with an unfavorable prognosis in CNS tumors [20]; thus, accurate analysis of markers from low-purity tumors is essential. In an analysis of 2249 tumors, 2188 (97.3 %) were found to have a purity above 50 % [20], resulting in a possible VAF> 25 % in case of heterozygous mutations. With a demonstrated sensitivity of 25 % VAF, our method has shown that heterozygous mutations can be reliably detected, even when considering tumor purity of ∼50 %, tumor heterogeneity, and potentially in biopsies obtained from tumor margins.

Due to the significant time involved in target amplification, the most suitable markers for this approach are single nucleotide variants (SNVs) located in well-defined, conserved regions of marker genes, allowing for the minimization of required amplicon lengths. For our approach, an amplicon length of 489 bp was enough to be used as input for the nanopore rapid sequencing kits SQK-RAD004 and SQK-RAD114, and it may not be the lower limit to feasible amplicon length. Shortening the amplicons for each marker could decrease both amplification and sequencing time, allowing for even faster time-to-results.

Challenges concerning a multiplex PCR for target sequence amplification include differences in primer affinities to their complementary sequences in terms of GC-content and gene region topology between amplification systems. These factors can lead to unequal amplification efficiencies among different primer pairs, resulting in imbalanced amplification of different target sequences. In the case of *pTERT*, which has a relatively high GC-content of 77.9 %, the amplification process may be further complicated, limiting the speed of multiplex time-to-results due to lower sequencing efficiency (Fig. 2, Fig. 3). Additionally, the topology of the binding region can influence primer annealing and amplification efficiency. These challenges underscore the importance of optimizing multiplex PCR conditions to ensure uniform amplification across all target sequences, especially in complex genomic regions such as *pTERT*.

An alternative amplification method could improve time-to-results, as PCR is responsible for 30 min of the overall time-to-results. The use of an isothermal amplification system such as Rolling Circle Amplification (RCA), Loop Mediated Isothermal Amplification (LAMP) or Recombinase Polymerase Amplification (RPA) [22] could provide an alternative to PCR for the preparation of next-generation amplicon sequencing libraries. Additionally, the time required for sequencing is inversely related to the speed of sequence acquisition. In addition to library quality and overall amplicon concentration, pore count is a determining factor for read acquisition when utilizing nanopore sequencing. Flongle flow cells possess a maximum of 126 channels (comprised of 4 pores on each channel), whereas MinION flow cells have an increased channel count of 512. Both flow cell types can be run using a MinION sequencer. The use of a MinION (R10.4.1) instead of a Flongle (R9.4.1) flow cell decreased the time required to pass the established pTERT read count threshold from 38:30 ± 17:41 min (or 37:34 ± 18:51 min for C228 and C250, respectively) to 20 min using isolated patient gDNA in the clinical demonstrator as the PCR template. To further improve the read speed, a PromethION flow cell with 2675 channels could be used for sequencing. The switch to a PromethION flow cell would require an investment in a PromethION sequencer, and thus, the benefit in sequencing speedup must be evaluated in terms of cost-effectiveness.

We note the limitations of intraoperative single nucleotide variant detection, as these markers cannot be used in isolation to unambiguously classify CNS malignancies. Markers such as IDH1 R132, IDH2 R172, H3F3A K27 and G34, and HIST1H3B K27 are associated with distinct methylation-based tumor classes [1], while BRAF V600E and pTERT C228T/C250T have been observed in various types of glioma [16] and other types of cancer [23]. DNA methylation profiling has emerged as a potentially immensely powerful technology for intraoperative CNS tumor classification. While intraoperative analysis of methylation patterns has shown significant advances and provides rapid diagnostic insights, it is not completely error-free and always depends on the secondary effect of a methylome alteration primarily caused by a mutation. The integration and parallel use of additional technologies, such as the shown mutation analysis, which shows the initial cause of the methylome alteration, can support the established whole genome approach and thus enhance the overall reliability and accuracy of intraoperative diagnostics.

Therefore, amplicon sequencing of tumor marker genes might be used in conjunction with other sequencing-based diagnostic workflows already established. For example, workflows such as Sturgeon aim to classify tumors based on methylome data, most effectively captured on PromethION flow cells [24]. The PromethION’s approximately four-fold throughput, when compared to a MinION system, could also be relevant for the intraoperative detection of copy number variations [25]. The low amount of input gDNA required (∼5 ng) for the PCR during the presented amplicon sequencing workflow could enable side by side execution with whole genome sequencing-based methylome analyses like Sturgeon (∼600 ng input gDNA required for library prep [7]). As methylome analysis detects the secondary effect of underlying genetic aberrations, the direct observation of genetic mutations of interest via SNV analysis possibly benefits tumor classification. The time-to-results of < 2 h for both workflows might enable a timely combination of methylome-based classifications with SNV data. Therefore, a combined approach of whole genome sequencing to assess methylome and copy number variations, as well as amplicon sequencing to gain insight into the mutational status of relevant prognostic and diagnostic tumor markers, might be a feasible and holistic approach to CNS tumor classification.

We envision the possibility of a single trained technician preparing the PCR and whole genome sequencing based setups in parallel. Sequencing could be performed on a single flow cell, if the rapid amplicon library is used to spike the WGS library. However, further investigations of these use cases would be required.

In summary, we show that multiplex amplicon sequencing can yield a direct tumor marker readout even for tumors of low purity in the timespan of typical CNS tumor resection surgery, allowing timely adaptation of the surgical strategy.

## 4 Materials and methods

### 4.1 Design of amplification systems

A range of pUC57 vectors containing a 2 kb insert of the relevant tumor marker genes *IDH1, IDH2, pTERT, H3F3A, Hist1H3B,* and *BRAF* were used as artificial templates for amplification. Amplicons were designed to cover conserved mutational hotspots of canonical transcripts of *IDH1, IDH2, pTERT, H3F3A, Hist1H3B* and *BRAF*. PCR primer sets for *IDH1, IDH2, pTERT* and *H3F3A* were drawn from previous works [4] and additional primer sets for *Hist1H3B* and *BRAF* were designed using Clone Manager 10 with default parameters to generate amplicons of similar lengths for multiplex compatibility (Supplementary Table 3). Amplicon sizes varied from 489 to 1098 bp (Supplementary Table 4).

### 4.2 Target amplification

A total of 8 ng of plasmid DNA, 13 ng of gDNA (gDNA-derived amplicon sequencing) and 5 ng of gDNA (Clinical Demonstrator) were used as templates for amplicon sequencing. PCR was performed using 0.02 U/µL Phusion HS II (Thermo Fisher), 250 µM dNTPs (BTR), 0.5 µM forward and reverse primer, 1x provided Phusion HS II high GC buffer and 5 % v/v DMSO in a total reaction volume of 100 µL, which were subsequently split into 5 × 20 µL. The following PCR protocol was performed on an Analytik Jena Biometra TAdvanced Twin Thermal Cycler: initial denaturation at 98 °C for 30 s; 25 cycles of 5 s of denaturation at 98 °C, 10 s of annealing at 59 °C and extension at 72 °C for 20 s; and a final extension for 300 s at 72 °C. The aliquots were pooled again. PCR products were purified using AMPure XP Beads (Beckman Coulter) according to the manufacturer’s protocol. Gel electrophoresis was performed on a 1 % agarose gel to confirm the amplification visually.

### 4.3 Amplicon sequencing

The generated purified amplicons (50–200 ng) were used for library preparation with a rapid sequencing kit (for Flongle flow cells: SQK-RAD004; for MinION flow cells: SQK-RAD114, Oxford Nanopore Technologies (ONT)) using modified manufacturer’s protocols (amplicon DNA instead of high molecular weight DNA; all volumes were halved to reduce library volume and therefore waste, when sequencing on Flongle flow cells). Preparation of flow cells and loading were performed according to the manufacturer’s protocols. Between 50 and 200 ng of amplicon DNA was used as input, and the resulting library was sequenced on Flongle R9.4.1 or MinION R10.4.1 flow cells (ONT). Fast5 files were basecalled in real time using guppy (build version 6.3.5) and further investigated *in silico.*

### 4.4 Python scripts

*In silico* analysis of the obtained sequences was performed using minimap2 (version 2.24) for alignment of the raw reads to modified target sequences. These target sequences had one or more nucleotides at the position of the SNV of interest removed. Variant calling of the alignment therefore revealed an artificial insertion at the putative mutation site. This was done to capture wild-type sequences as well as variants during variant calling for increased accuracy. Variant calling was performed using PAFtools (version 2.24). The artificial insertions found were tallied by custom python scripts, classified as wild-type, mutant or unrecognizable and visualized via MatplotLib. All custom scripts used for identifying and tallying tumor marker variants can be found here: https://github.com/maximilianevers/AmpliSeq.

### 4.5 Intraoperative tumor DNA extraction

After reception of the tumor tissue, gDNA was extracted using the QIAamp Fast DNA Tissue Kit (Qiagen). 15 mg of brain tumor tissue was transferred into a tissue disruption tube and lyzed in 265 µL of digestion buffer mix using the TissueLyser LT (Qiagen) for 2 min at 45 Hz. Afterwards, protein and RNase digestion were performed at 56 °C for 7 min and 1000 rpm in a Thermomixer (Eppendorf). Then, 265 µL of MVL buffer was added to the sample, which was subsequently homogenized by pipetting. The precipitated DNA mixture was purified via a QIAamp mini spin column. Centrifugation was performed at 20,000 × *g* for 1 min. Two wash steps were carried out with 500 µL of buffer AW1 and AW2, followed by centrifugation at 20,000 × *g* for 30 s. Residual ethanol was removed via centrifugation at 20,000 × *g* for 2 min. DNA was eluted in 50 µL of preheated (56 °C) nuclease-free H_2_O for 1 min, followed by centrifugation at 20,000 × *g* for 1 min. A Nanodrop One (Thermo Fisher Scientific) was used for DNA quantification. The extracted DNA was stored at −80 °C before library preparation.

### 4.6 Sturgeon classification

To prepare rapid libraries, the SQK-RAD004 Sequencing Kit (ONT) was used following a modified manufacturer’s protocol. A total of 600–700 ng of extracted gDNA was adjusted to a total volume of 7.5 µL with nuclease-free H_2_O. Then, 2.5 µL of fragmentation mix (FRA) was added to the sample and immediately incubated at 30 °C for 1 min using a preheated thermocycler (Doppio, VWR). Afterwards, a heat inactivation step was performed at 80 °C for 1 min. 1 µL of rapid adapter (RAP) was added to the mix before incubation at RT for 10 min. MinION flow cells (ONT, FLO-MIN106D R9.4.1) were primed following the manufacturer’s protocol. To the libraries, 34 µL of sequencing buffer (SQB), 25.5 µL of loading beads and 4.5 µL of nuclease-free water were added before they were loaded onto the flow cells.

Sturgeon classifications were generated each minute during the first 60 min of sequencing. The results with scores lower than 0.8 were considered inconclusive.

## Ethical considerations

Samples were collected from the Department of Neurosurgery of the University Medical Center, UKSH, Kiel, Germany after obtaining the written informed consent of the donors for diagnostic procedures. The study was approved by and adhered to the Ethics Committee of the University of Kiel (D 443/20) and is in accordance with the Helsinki Declaration of 1975 and its further amendments.

## CRediT authorship contribution statement

**Stephan Kolkenbrock:** Writing – review & editing, Supervision, Project administration, Methodology, Funding acquisition, Conceptualization. **Sönke Friedrichsen:** Writing – review & editing, Methodology, Funding acquisition, Conceptualization. **Gaojianyong Wang:** Writing – review & editing, Methodology. **André Maicher:** Writing – review & editing, Methodology, Conceptualization. **Dominik Danso:** Writing – review & editing, Methodology, Conceptualization. **Franz-Josef Müller:** Writing – review & editing, Resources, Methodology, Conceptualization. **Carolin Kubelt-Kwamin:** Writing – review & editing, Resources. **Christian Rohrandt:** Software, Methodology. **Björn Brändl:** Methodology, Data curation. **Maximilian Evers:** Writing – original draft, Visualization, Software, Methodology, Investigation, Data curation.

## Declaration of Competing Interest

All authors declare that they have no conflicts of interest.
