# Supplementary material for: Rapid intraoperative amplicon sequencing of CNS tumor markers
Source: Comput Struct Biotechnol J. 2024 Nov 8;26:51–7. doi: 10.1016/j.csbj.2024.11.007 (PMC11600771; doi:10.1016/j.csbj.2024.11.007)
Supplement: Supplementary file 1 — Supplementary material [file mmc1.docx]

**Supplementary Materials**

Supplementary Figure 1 – Results of amplicon sequencing runs investigating a marker panel consisting of IDH1, IDH2, pTERT, H3F3A, Hist1H3B and BRAF. Allele frequency [%] for each investigated marker variant is given. Wildtype allele frequency is indicated in shades of green. Variant allele frequency is indicated in shades of red. If present, routine diagnostic results are indicated. Clinically relevant data is given as available.


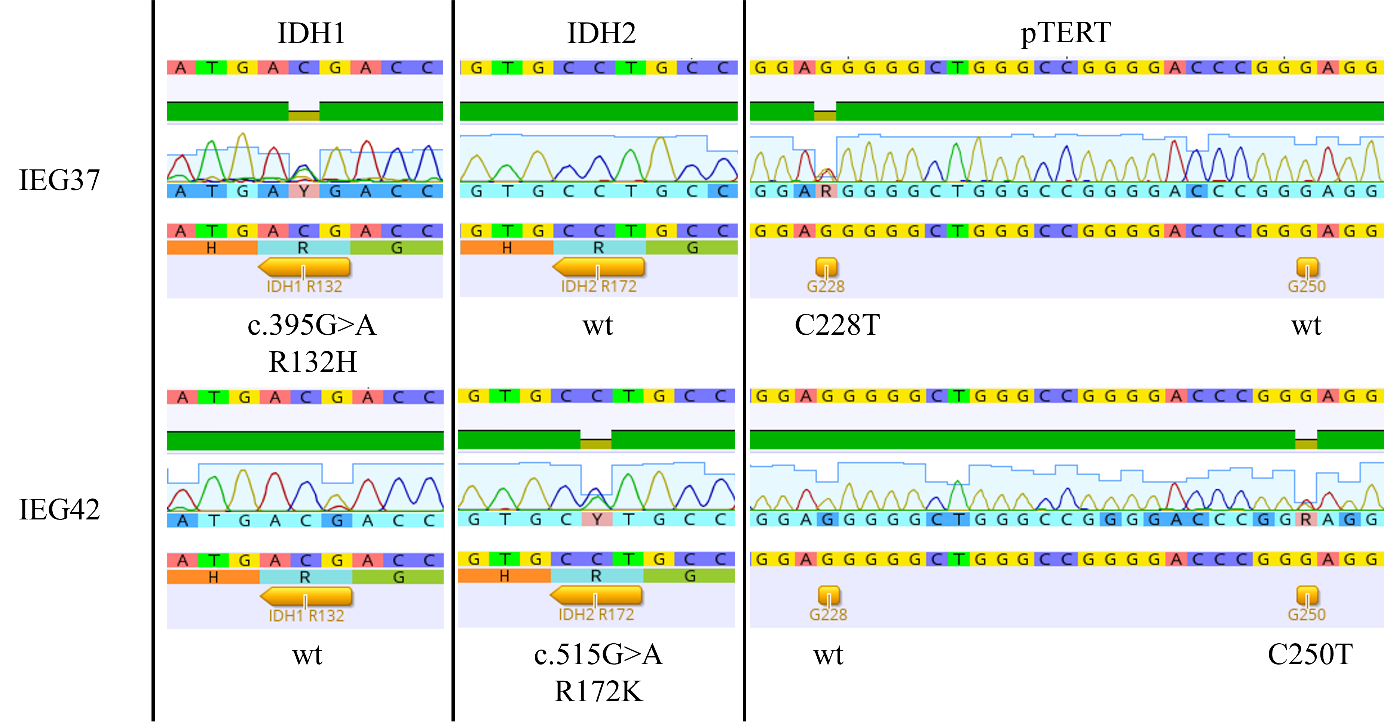


Supplementary Figure 2 – Confirmed heterozygous mutations in samples IEG37 (IDH1 R132H; pTERT C228T) and IEG42 (IDH2 R172K; pTERT C250T) via Sanger sequencing. The same primers were used for sequencing and amplification. The ab1 files generated by Sanger sequencing are displayed in Geneious 10.2.6.


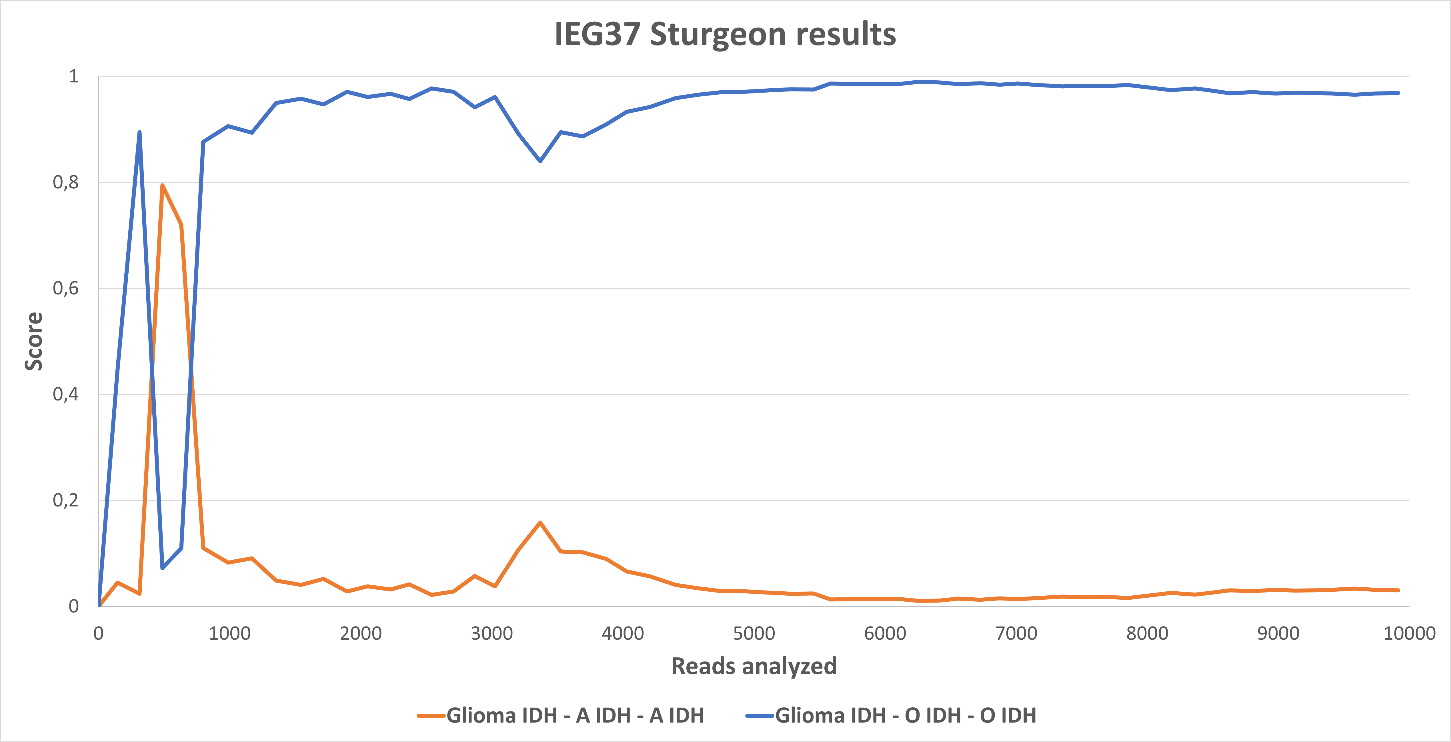


Supplementary Figure 3 – Sturgeon classification for sample IEG37. The two most prominent classifications (of 87 sturgeon classes) are shown. Sturgeon classification suggests an oligodendroglioma harboring an IDH mutation.


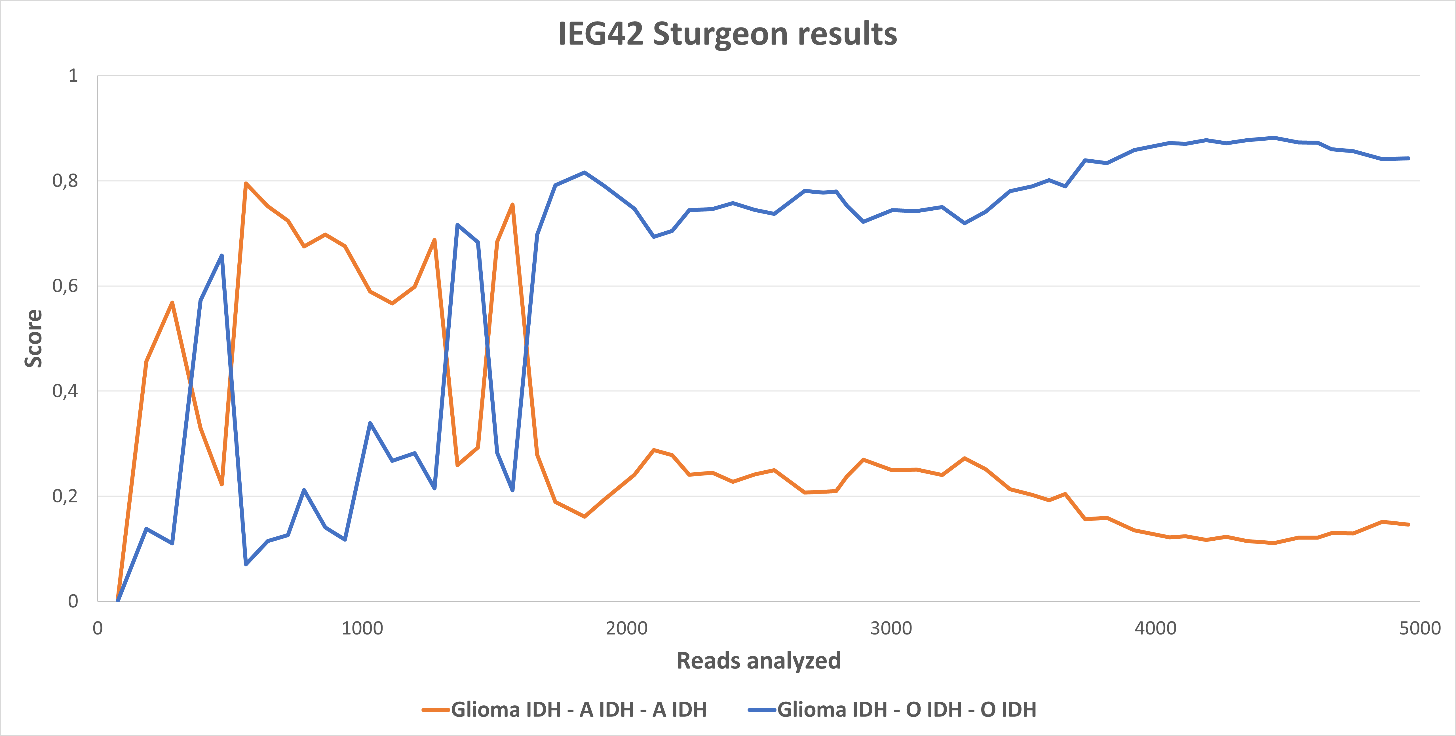


Supplementary Figure 4 – Sturgeon classification for sample IEG42. The two most prominent classifications (of the 87 sturgeon classes) are shown. Sturgeon classification suggests an oligodendroglioma harboring an IDH mutation.


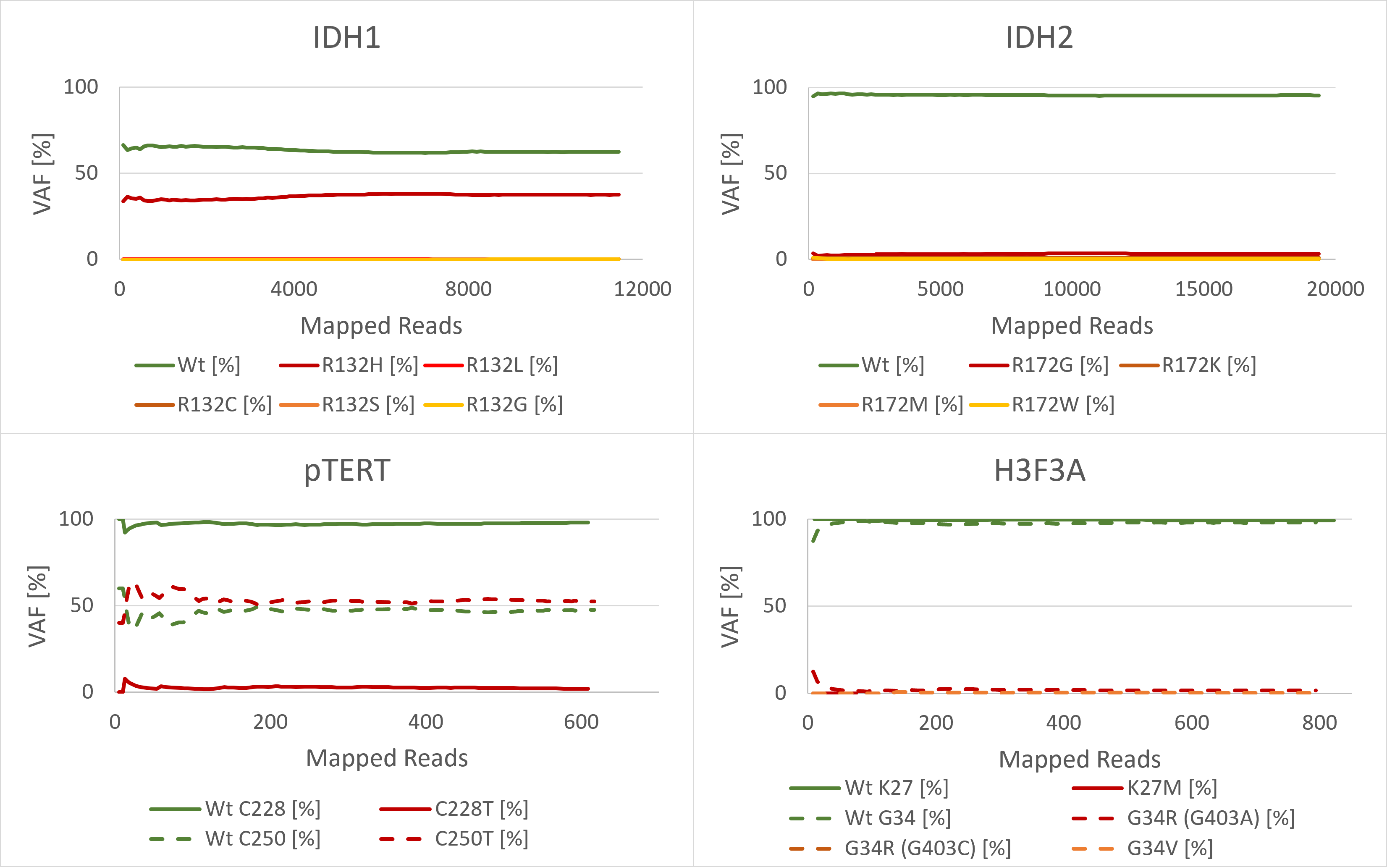


Supplementary Figure 5 – Results of first clinical demonstrator using a marker panel of IDH1, IDH2, pTERT C228 and C250, H3F3A K27 and G34. Results after 3h of sequencing on MinION Flowcell R9.4.1. Library was prepared using Ligation Sequencing Kit (SQK-LSK109).

Supplementary Table 1 – Summary of mapped sequences generated during the first 3 h of sequencing of the first clinical demonstrator. Only reads that were mapped to a specific marker and contained information about the SNV of interest are shown.

| **Marker** | **Reads** | **Reads [%]** |
| --- | --- | --- |
| **IDH1 - All** | **11443** | **100.00** |
| Wt | 7136 | 62.36 |
| R132H | 4296 | 37.54 |
| R132L | 0 | 0.00 |
| R132C | 5 | 0.04 |
| R132S | 4 | 0.03 |
| R132G | 2 | 0.02 |
| **IDH2 - All** | **19357** | **100.00** |
| Wt | 18475 | 95.44 |
| R172G | 628 | 3.24 |
| R172K | 189 | 0.98 |
| R172M | 7 | 0.04 |
| R172W | 58 | 0.30 |
| **pTERT C228 - All** | **609** | **100.00** |
| Wt | 597 | 98.03 |
| C228T | 12 | 1.97 |
| **pTERT C250 - All** | **617** | **100.00** |
| Wt | 294 | 47.65 |
| C250T | 323 | 52.35 |
| **H3F3A K27 - All** | **822** | **100.00** |
| Wt | 816 | 99.27 |
| K27M | 6 | 0.73 |
| **H3F3A G34 - All** | **794** | **100.00** |
| Wt | 778 | 97.98 |
| G34R (G403A) | 14 | 1.76 |
| G34R (G403C) | 0 | 0.00 |
| G34V | 2 | 0.25 |

Supplementary Table 2 – Summary of mapped sequences generated during the first 20 min of sequencing of the second clinical demonstrator. Only reads that were mapped to a specific marker and contained information about the SNV of interest are shown.

| **Marker** | **Reads** | **Reads [%]** |
| --- | --- | --- |
| **IDH1 - All** | **3388** | **100.00** |
| Wt | 3156 | 93.15 |
| R132H | 82 | 2.42 |
| R132L | 0 | 0.00 |
| R132C | 16 | 0.47 |
| R132S | 71 | 2.10 |
| R132G | 63 | 1.86 |
| **IDH2 - All** | **2967** | **100.00** |
| Wt | 2932 | 98.82 |
| R172G | 18 | 0.61 |
| R172K | 14 | 0.47 |
| R172M | 3 | 0.10 |
| R172W | 0 | 0.00 |
| **pTERT C228 - All** | **94** | **100.00** |
| Wt | 94 | 100.00 |
| C228T | 0 | 0.00 |
| **pTERT C250 - All** | **85** | **100.00** |
| Wt | 30 | 35.29 |
| C250T | 55 | 64.71 |
| **H3F3A K27 - All** | **1326** | **100.00** |
| Wt | 1315 | 99.17 |
| K27M | 11 | 0.83 |
| **H3F3A G34 - All** | **1506** | **100.00** |
| Wt | 1469 | 97.54 |
| G34R (G403A) | 37 | 2.46 |
| G34R (G403C) | 0 | 0.00 |
| G34V | 0 | 0.00 |
| **Hist1H3B - All** | **1857** | **100.00** |
| Wt | 1846 | 99.41 |
| K27M | 11 | 0.59 |
| **BRAF - All** | **2103** | **100.00** |
| Wt | 2093 | 99.52 |
| V600E | 10 | 0.48 |


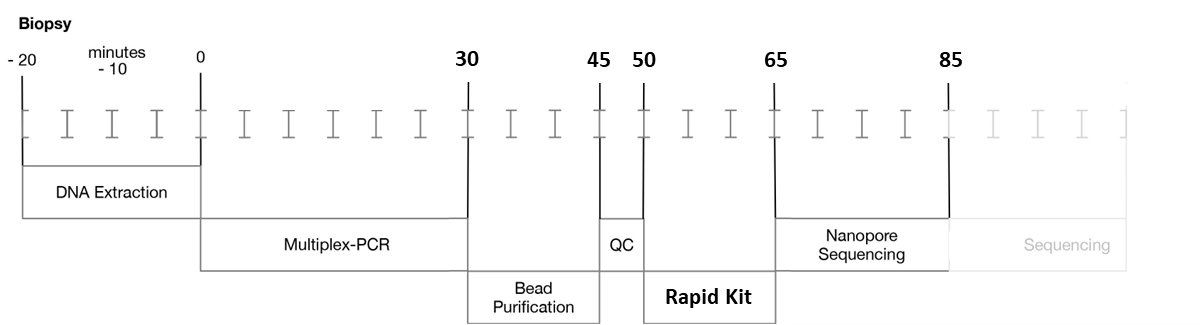


Supplementary Figure 6 – Method timeline for multiplex amplicon-based tumor marker sequencing.

Supplementary Table 3 –Primer systems used for amplification of different tumor markers.

| **Target** | **Fw-primer** | **Rv-primer** |
| --- | --- | --- |
| *IDH1* | ATGTGTTGAGATGGACGCCT | TCCCACCACTTCAACGTCAA |
| *IDH2* | AGTGTGTCCCTGGCTTATCC | ACTCCAGAGCCCACACATTT |
| *pTERT* | GGCCGATTCGACCTCTCT | CCACTACCGCGAGGTGCT |
| *H3F3A* | CAAAGGCCGTTCGAGGTATT | ACAGTGGCTCAGGTAGTTCA |
| *Hist1H3B* | ATTGGAGGTTTCCGCGTTGC | TTACGCTCTTTCTCCGCGAAT |
| *BRAF* | GCAAGACAGCACAAGGCTGTTA | TGAAGAGCCTTTACTGCTCGC |

Supplementary Table 4 – Genomic start and end coordinates (GRCh.38) of analyzed amplicons, amplicon length and analyzed single nucleotide variants (SNV) within the tumor marker sequences.

| **Target** | **Start** | **End** | **Length [bp]** | **SNV** |
| --- | --- | --- | --- | --- |
| *IDH1* | 2:208248152 | 2:208249113 | 962 | rs121913500; rs121913499 |
| *IDH2* | 15:90087910 | 15:90088899 | 990 | rs1057519906; rs121913503 |
| *pTERT* | 5:1294925 | 5:1295413 | 489 | rs1242535815; rs990029964 |
| *H3F3A* | 1:226064051 | 1:226064964 | 914 | rs1057519903; rs1553260624; rs2102735743 |
| *Hist1H3B* | 6:26032373 | 6:26031457 | 916 | rs2113678877 |
| *BRAF* | 7:140752642 | 7:140753740 | 1098 | rs113488022 |
